# Optimising the use of mTOR inhibitors in renal transplantation

**DOI:** 10.1186/2047-1440-2-S1-S4

**Published:** 2013-11-20

**Authors:** Graeme R Russ

**Affiliations:** 1 Central and Northern Adelaide Renal and Transplantation Service, Royal Adelaide Hospital, North Tce, Adelaide, Australia 5000; 2 Faculty of Health Sciences, University of Adelaide, North Tce, Adelaide, Australia 5000; 3 ANZDATA Registry, Royal Adelaide Hospital, North Tce Adelaide, Australia 5000

**Keywords:** kidney transplant, mammalian target of rapamycin inhibitors, immunosuppression

## Abstract

Renal transplantation is the treatment of choice for end-stage renal failure. Although advances in immunosuppression have led to improvements in short-term outcomes, graft survival beyond 5 to 10 years has not improved. One of the major causes of late renal allograft failure is chronic allograft nephropathy, a component of which is nephrotoxicity from the use of calcineurin inhibitors (CNIs). In addition, premature patient death is a major limitation of renal transplantation and the major causes are cancer, cardiovascular disease and infection. CNI-free immunosuppressive regimens based on mammalian target of rapamycin (mTOR) inhibitors have been trial led over the last few years and have defined the rational use of these agents. Conversion from a CNI-based to an mTOR-inhibitor-based regimen has been successful at improving renal function for a number of years after conversion, although long-term survival outcomes are still awaited. The studies suggest that the safest and most effective time to convert is between 1 and 6 months after transplant. In addition, mTOR-inhibitor-based regimens have been shown to be associated with lower rates of post-transplant malignancy and less cytomegalovirus infection, which may add further to the appeal of this approach.

## Long-term outcomes in renal transplantation

The aim of offering renal transplantation to patients with end-stage renal failure is to achieve a longer and better quality of life than can be achieved with dialysis therapy. For most patients this is being achieved, but the long-term results come nowhere near what could be expected for the normal age-matched population. The Australia and New Zealand Dialysis and Transplant Registry (ANZDATA) shows that for patients transplanted with a primary deceased donor graft from 1995 to 2000 in Australia and New Zealand, 72% were alive 10 years after transplantation [1]. Of these, 20% had returned to dialysis therapy. Only 59% of these patients were thus alive with a functioning graft after 10 years.

The death rate after the first year for this cohort of patients is 2.5% per year. Deaths in the first few years relate to infection, with an increasing proportion due to malignancy and cardiovascular disease in later years. In 2010 in Australia, 32% of deaths in transplant patients were due to malignancy, compared with 23% from cardiac or vascular disease and 22% from infection [1].

**Figure 1:**
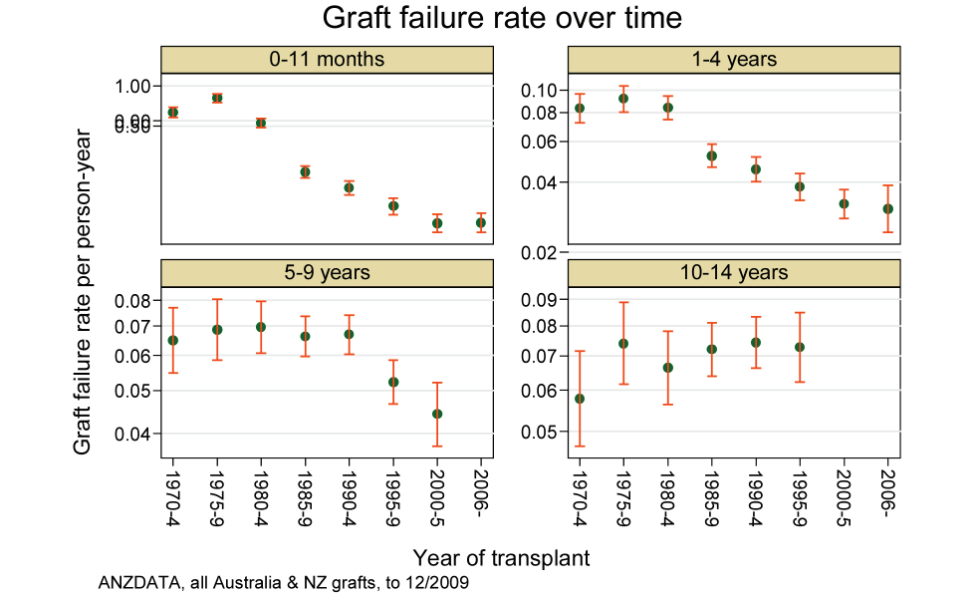
**Graft failure rates per person-year for all renal transplants in Australia and New Zealand, 1970 to 2009.** Graphs show rates by time after transplantation for era from 1970 divided into 5-year blocks [1]. ANZDATA, Australia and New Zealand Dialysis and Transplant Registry; NZ, New Zealand.

Whereas improvements in immunosuppression and surgical and medical advances have resulted in short-term gains, the long-term outcomes have not improved. To illustrate this fact we have analysed ANZDATA for graft failure rates for transplants performed between 1970 and 2009. Over those decades the graft failure rates have fallen substantially for the first year and to a lesser degree for the time periods of 1 to 4.9 years and 5 to 9.9 years. For those grafts surviving 10 years, however, the subsequent annual failure rate has increased over time (Figure 1).

## Role of immunosuppression in long-term outcomes

The role of chronic immunosuppression in adverse patient outcomes has long been recognised. The contribution to mortality from infection is well known. In addition, the worsening of metabolic abnormalities such as diabetes and hyperlipidaemia and elevations in blood pressure contribute to cardiovascular risk. Immunosuppression in combination with other oncogenic stimuli such as viruses and ultraviolet radiation causes significant increases in the risk of development of certain cancers such as Kaposi sarcoma, lymphoma and nonmelanotic skin cancers.

Different immunosuppressive drugs exhibit different profiles of toxicity and may influence in different ways the adverse outcomes seen in the long term. Much attention has been directed to the contribution of chronic nephrotoxicity of calcineurin inhibitors (CNIs) to long-term graft failure. A study of protocol biopsies of renal allografts from 3 months to 10 years after transplantation by Nankivell and colleagues described the development of chronic allograft nephropathy (CAN) over time [2]. When changes of CAN – in particular, interstitial fibrosis and tubular atrophy – developed in the first year after transplantation, these were attributed to early events such as rejection and acute tubular necrosis. Lesions such as arteriolar hyalinosis and glomerulosclerosis developed later and were said to indicate damage from cyclosporine exposure.

Recently, doubt has been cast on the relative contribution of CNI nephrotoxicity to long-term graft failure relative to other causes of graft damage such as T-cell-mediated and antibody-mediated rejection, polyoma virus infection and recurrent glomerulonephritis. In a North American study of biopsies of failing grafts, the dominant histological lesion seen at 5 years was chronic antibody-mediated rejection [3]. However, most of the biopsies were performed in response to deteriorating renal function or other clinical events at a median time of 17 months after transplantation. This study therefore differs from the study by Nankivell and colleagues, where the biopsies were performed at protocol-derived time points to 10 years.

There are very few studies where the hard endpoints of graft loss or patient death give an indication of the adverse effects of long-term CNI therapy. One of these studies is the Australian Multicentre Cyclosporine Renal Transplant Study, which has recently published results after 20 years of follow up [4]. Patients randomised to ongoing cyclosporine A (CsA) had an inferior outcome compared with patients who received CsA for 3 months before converting to azathioprine and corticosteroid. This study therefore supports that CNI exposure contributes to deteriorating renal function and graft loss in the very long term after renal transplantation. Moreover, the study demonstrates that the effect of CNI nephrotoxicity may not be clinically evident until the second decade after transplantation.

Much has also been made recently of the nonspecific nature of the histopathological lesions purported to indicate evidence of CNI nephrotoxicity [5]. Whereas many investigators concede that the lesions of arteriolar hyalinosis occur with chronic CNI toxicity, they are present in many other renal pathological conditions and may also be present at the time of transplantation as a result of donor-related disease. A recent study from France has demonstrated that the lesions described by Nankivell and colleagues as being characteristic of CAN and chronic CNI toxicity are seen in patients who have not been exposed to a CNI [6]. However, when comparing biopsies from patients who were receiving CNIs and those on sirolimus (SRL), the lesions of CNI arteriolopathy were more common in those patients receiving CNI therapy.

## Rationale for mammalian target of rapamycin-inhibitor use in renal transplantation

Mammalian target of rapamycin (mTOR) inhibitors have been shown to be effective immunosuppressant agents in renal transplantation either in combination with a CNI or not. The main rationale for using these agents in a CNI-free regimen is to avoid the adverse events associated with CNI use, in particular chronic nephrotoxicity. There is, however, accumulating evidence that the avoidance of CNIs and the use of mTOR inhibitors also confer benefits with respect to the development of malignancy and also some post-transplant infections.

## Effect of mTOR inhibitors on post-transplant renal function

The first study to demonstrate the beneficial effect of long-term CNI-free mTOR-inhibitor-based therapy in renal transplantation was the Rapamune Maintenance Regimen (RMR) study [7]. Patients were randomised at 3 months to either continue a regimen of SRL, CsA and steroids, or to have CsA withdrawn with an increase in the concentration of the targeted dose of SRL. Although 525 patients entered this study, 95 were not randomised because of significant rejection in the first 3 months or because of poor transplant function. The randomised patients were therefore a group selected for favourable outcomes. Nevertheless, in spite of having numerically greater numbers of rejections in the 3 months after randomisation, at 4 years the patients in whom CsA was withdrawn had superior renal function and graft survival compared with those who continued the combination of CsA and SRL. In addition, protocol biopsies performed at 36 months showed less CAN in the CsA withdrawal group [8]. One valid criticism of this study is that the control arm of CsA and SRL is more nephrotoxic and would therefore provide an unfair comparator for both renal function and structure.

From *in vitro* experimental studies it has long been recognised that the combination of a CNI and an mTOR inhibitor provide immunological synergy. However, the main limitation of this combination in clinical practice is the enhanced nephrotoxicity of the CNI. Randomised trials using everolimus with a reduced dose of CsA have nevertheless demonstrated that efficacy is maintained without any detriment to renal function, at least at the relatively early time point of 24 months [9]. This approach has allowed a 60% reduction in exposure to the CNI over a 12-month timeframe. The longer term effect of this approach on renal function is not known and awaits further observation.

Other studies have used mTOR inhibitors as *de novo* therapy without concomitant CNI. The ORION study was a three-arm randomised controlled trial in which patients who received SRL, mycophenolate mofetil (MMF), steroid and basiliximab had a higher rate of acute rejection at 6 months compared with patients receiving a similar regimen but with tacrolimus rather than SRL [10]. In the Symphony study, patients were randomised to one of four treatment groups: MMF with standard-dose CsA and corticosteroids; MMF with low-dose CsA, daclizumab and corticosteroids; MMF with low-dose tacrolimus, daclizumab and corticosteroids; or MMF with low-dose SRL, daclizumab and corticosteroids [11]. This study found that the regimen containing low-dose tacrolimus resulted in improved renal function, graft survival, and acute rejection rates compared with SRL/MMF and the other regimens, and that this was sustained over 3 years of follow up [12]. Even though the blood concentrations of SRL in these studies may have been lower than optimal, SRL/MMF would appear to be a less potent immunosuppressive combination than CNI/MMF, especially in the first few months after transplant when rejection is more likely to occur. A 2011 meta-analysis assessing outcomes associated with reducing CNI exposure from the time of transplantation found that there was no difference in acute rejection rates with mTOR inhibitors and MMF in combination compared with CNI-based regimens (16 studies, *n* = 2,688) [13]. Use of an mTOR-inhibitor/MMF combination immediately following transplant was associated with improved graft function but was also associated with increased graft failure, suggesting that the benefit of improved renal function is offset by increased graft loss [13].

The fact that long-term SRL without CNI showed excellent outcomes in terms of renal function at 5 years in the RMR study has prompted investigators to convert patients from a CNI to an mTOR inhibitor at varying times after transplantation with the aim of improving graft function. The CONVERT study examined late conversion, approximately 3 years after transplantation, from a CNI to SRL [14]. Two years after conversion, renal function improved (slightly but not significantly) in patients with good transplant function (glomerular filtration rate (GFR) >40 ml/minute). Inferior outcomes were seen in those with poorer function or significant proteinuria [14].

More recent studies have been published where conversion has occurred earlier, and in general these approaches have been associated with greater benefit to renal function. The CONCEPT study is a randomised controlled trial from France demonstrating that conversion at 3 months from CsA to SRL in a regimen of CsA, mycophenolate, steroids and daclizumab leads to a clinically significant improvement in renal function without any detriment to graft or patient survival at 12 months [15]. There was an increase in the rejection rate in the conversion arm but this only occurred after steroids were withdrawn by protocol at 8 months [15]. Recently, the improvement in renal function has been demonstrated to be maintained to 5 years, with an approximately 10 ml/minute greater estimated GFR in the conversion group [16]. Similarly, the Spare-The-Nephron study from the USA randomised patients on a CNI/mycophenolate/steroid regimen to conversion to a SRL/mycophenolate/steroid regimen 1 to 6 months after transplantation [17]. At 1 year, the measured GFR in the converted group had significantly improved by 24% compared with 5% in the control. The difference between groups was maintained at 2 years. Similar results have been obtained when a CNI is converted to everolimus. The ZEUS study randomised patients to conversion at 4.5 months from CsA to everolimus in combination with mycophenolate, steroids and induction with basiliximab [18]. At 1 year, the rejection rates were the same although there were numerically more episodes in the everolimus patients in the few months after conversion. There was, however, approximately a 10 ml/minute higher GFR in the patients who converted to everolimus.

A meta-analysis of 22 randomised controlled trials assessed outcomes following conversion from CNIs to mTOR inhibitors for maintenance immunosuppression [19]. The analysis found that, compared with CNI-based regimens, mTOR-inhibitor-based regimens were associated with improved renal function (estimated GFR) at 12 months and this was sustained 5 years post transplant. When results at 12 months were stratified by time to conversion, early conversion (<12 months post transplant) was found to result in improved graft function compared with later conversion. The estimated GFR (expressed as weighted mean difference) was 5.07 ml/minute for early conversion, and was 2.85 ml/minute for later conversion. mTOR inhibitors were associated with a higher rate of rejection in the first 12 months, but there was no difference in rejection after 12 months. mTOR inhibitors were also associated with a lower risk of graft loss between 2 and 5 years post conversion. The risk of adverse events, such as oedema, proteinuria and hyperlipidaemia, was higher for mTOR inhibitors, but infection rates were similar.

There are two notable observations from these studies. The first is that rejection episodes occur after conversion in about 5 to 10% of patients. From the CONCEPT study, it would appear that there is a greater chance of rejection if steroids are withdrawn from an mTOR-inhibitor/MMF combination. These rejection episodes are usually mild and easily reversible. A number of investigators have observed that it is critical to maintain adequate blood concentrations of mTOR inhibitors to reduce the rate of rejection. The second observation is that the tolerability of the mTOR inhibitor is such that up to 25% of patients return to the CNI-based combination. The usual reasons for poor tolerability are mouth ulcers, oedema, and dyslipidaemia.

The above studies suggest that renal graft function is better if conversion from a CNI to an mTOR inhibitor is performed between 1 and 6 months post transplant; and for patients who tolerate the mTOR inhibitor, this improvement is maintained for at least 2 years and possibly 5 years after conversion.

## Effect of mTOR inhibitors on the development of post-transplant malignancy

There are firm theoretical and experimental reasons why mTOR inhibitors might protect against the development of malignancy. There is now clinical evidence that patients treated with SRL have a lower rate of post-transplant malignancy compared with those receiving CNI-based regimens [20]. In addition, mTOR inhibitors have shown further anti-cancer properties with promising results in the treatment of nontransplant patients with B-cell lymphomas and have been trialled in the treatment of relapsed mantle cell lymphoma [21].

Initially, registry analysis from the USA showed a lower incidence of both skin and solid organ malignancy in patients taking mTOR inhibitors (alone or in combination with CNIs) compared with patients on a CNI-based regimen [22]. Although they were not designed to evaluate cancer outcomes, the RMR study [23], the CONVERT study [24] and the CONCEPT study [16] all showed a reduced rate of malignancy development after conversion from a CNI to an mTOR inhibitor. This reduction was true for both skin and solid organ malignancies. In the RMR study, patients who received SRL-based CNI-free immunosuppression experienced a significantly reduced risk of both skin cancer and nonskin cancer at 5 years after transplantation compared with those who received SRL/CNI combination therapy [23]. In the CONVERT study, the rate of cancer development was approximately one-third in those patients converted to SRL at 2 years after conversion [24].

The only randomised controlled trial reported to date that has assessed cancer development after conversion from a CNI to an mTOR inhibitor has been the 407 skin cancer study [25]. Performed predominantly in Australia, where the rate of post-transplant nonmelanotic skin cancer is high, 86 renal transplant patients were randomised to convert to SRL or to continue CNIs. The average time after transplant was almost 10 years, and tolerability of SRL was poor such that almost one-half of the converted patients did not maintain treatment with SRL. Follow up was for at least 1 year after conversion. Nevertheless, on analysis of the intention-to-treat population, the rate of nonmelanoma skin cancer development was 2.48 per patient-year in the group continuing CNIs compared with 1.31 in the group converted to SRL (*P* = 0.022).

Two trials have focused on secondary skin cancer prevention and have found conflicting results. In the RESCUE study [26] and the TUMORAPA study [27], patients with at least one biopsy-confirmed squamous cell carcinoma were randomly assigned to convert to SRL or to continue their original regimen. In the RESCUE study, results did not show a benefit of conversion to SRL in terms of squamous cell carcinoma-free survival at 2-year follow up [26]. In the TUMORAPA study, however, SRL demonstrated a significant anti-tumoural effect at 2 years with a decreased risk of new squamous cell carcinoma and longer time to development of new lesions [27].

Overall, the evidence thus suggests that conversion to SRL after transplantation is associated with a lower rate of malignancy development compared with continuation of CNIs, and that the difference is evident as early as 1 year after conversion, at least for patients at risk of skin cancer development.

## A lower rate of viral disease after transplantation with mTOR-inhibitor therapy

There is now convincing evidence that immunosuppressive regimens, including mTOR inhibitors, are associated with less cytomegalovirus infection and disease (reviewed in [28]). Rates of cytomegalovirus are lower with both SRL [15] and everolimus [9], in recipients of kidney [9,15] and heart [29] grafts, whether the mTOR inhibitor is used with or without a CNI, and whether or not anti-viral prophylaxis is used [30].

Rates of BK virus infection have also been shown to be lower with mTOR-inhibitor-based regimens, with data showing a lower rate of BK virus-related events in patients receiving everolimus or SRL [31].

## Effect of mTOR inhibitors on cardiovascular disease

Along with cancer, cardiovascular disease is one of the main causes of death with a functioning graft [1]. Immunosuppressants have differing effects on cardiovascular risk factors, such as hypertension, left ventricular hyperplasia, and dyslipidaemia, and selection of immunosuppression may be used to help control these risk factors. Conversion from CNI-based to mTOR-inhibitor-based immunosuppression has been shown to improve blood pressure and may achieve regression of left ventricular hyperplasia. In the RMR study, it was found that conversion to SRL resulted in significantly better mean arterial blood pressure at most time points from 6 to 48 months. Patients converted to SRL also received fewer anti-hypertensive drugs [7]. However, the group converted to SRL also had significantly higher levels of high-density lipoprotein cholesterol. Dyslipidaemia has been found to be more prevalent in patients converted to mTOR-inhibitor-based regimens, with increased levels of cholesterol and triglycerides, and an increased use of lipid-lowering agents [32]. Experimental and clinical studies have shown that SRL may be effective in achieving regression of left ventricular hyperplasia [33]; more trials are needed in this area.

## Conclusion

The benefit of a long-term CNI-free mTOR-inhibitor-based regimen after renal transplant is the promise of reduced development of chronic damage to the graft mediated by CNI, and a lower incidence of post-transplant malignancy. CNI-free mTOR-inhibitor-based regimens are less efficacious with respect to rejection prophylaxis and their use in the *de novo* transplant is not recommended. Conversely, when conversion from a CNI to an mTOR inhibitor is performed late, the patient has GFR <40 ml/minute, or the patient has pre-existing proteinuria, as in the CONVERT study, then benefit is blunted. The studies that have shown the greatest benefit in terms of renal allograft function are those where conversion is attempted between 1 and 6 months after transplantation. When patients have been able to tolerate a switch from a CNI to an mTOR inhibitor in this time period, the benefits in the medium term are better allograft function, a lower incidence of cancer, and possibly a lower rate of viral infection. The long-term impact (beyond 10 years) of this strategy requires well-designed trials with late follow up, which would only be possible using registries such as ANZDATA.

## Abbreviations

ANZDATA: Australia and New Zealand Dialysis and Transplant Registry; CAN: chronic allograft nephropathy; CNI: calcineurin inhibitor; CsA: cyclosporine A; GFR: glomerular filtration rate; MMF: mycophenolate mofetil; mTOR: mammalian target of rapamycin; RMR: Rapamune Maintenance Regimen; SRL: sirolimus.

## Competing interests

GRR has received honoraria from Pfizer, Novartis, Astellas and Janssen Cilag.

## Declaration

This article has been published as part of *Transplantation Research* Volume 2 Suppl 1, 2013: Proceedings of the 12th International Symposium on Transplantation. The full contents of the supplement are available at http://www.transplantationresearch.com/supplements/2/S1. The supplement is based on the 12th International Transplantation Symposium held on 16–17 April 2012, in Istanbul, Turkey. The symposium and publication based on these proceedings were supported by an unrestricted educational grant from Pfizer Inc., who had no influence on the editorial content. Editorial support was provided by Synergy, Richmond, UK and was funded by Pfizer Inc. Rapamune (sirolimus) is a product marketed by Pfizer Inc. The articles have been through the journal’s standard peer review process and the Supplement Editors declare that they have no competing interests.
